# Genetic Characterization of the RAP-1A and SBP-4 Genes of *Babesia* Species Infecting Cattle from Selangor, Malaysia, and Ribah, Nigeria

**DOI:** 10.3390/pathogens13030247

**Published:** 2024-03-13

**Authors:** Adamu Isah Gano, Siti Zubaidah Ramanoon, Nor-Azlina Abdul Aziz, Mazlina Mazlan, Mohd Rosly Shaari, Abdullahi Aliyu, Muhammad Bashir Bello, Mustapha Umar Imam, Hazilawati Hamzah

**Affiliations:** 1 Department of Veterinary Pathology and Microbiology, Faculty of Veterinary Medicine, University Putra Malaysia, Serdang 43400, Selangor, Malaysia; ganovet@yahoo.com (A.I.G.); azlinaaziz@upm.edu.my (N.-A.A.A.);; 2 Nigeria Agricultural Quarantine Service, Plot 84, Ralph Sodeinde Street, Central Business District, Abuja 900211, Nigeria; 3 Department of Farm and Exotic Animal Medicine and Surgery, Faculty of Veterinary Medicine, University Putra Malaysia, Serdang 43400, Selangor, Malaysia; 4 Animal Science Research Centre, Malaysian Agricultural Research and Development Institute, Headquarters, Serdang 43400, Selangor, Malaysia; 5 Department of Veterinary Medicine, College of Applied and Health Sciences, A’Sharqiyah University, P.O. Box 42, Ibra 400, Oman; abdullahi.aliyu@asu.edu.om; 6 Department of Veterinary Pathology, Faculty of Veterinary Medicine, Usmanu Danfodiyo University, Sokoto 840212, Sokoto State, Nigeria; 7 Centre for Advance Medical Research and Training, College of Health Sciences, Usmanu Danfodiyo University, Sokoto 840212, Sokoto State, Nigeria; 8 Infectious Disease Research Department King Abdullah International Medical Research Center, Riyadh P.O. Box 3660, Saudi Arabia

**Keywords:** babesia, cattle, nested PCR, phylogenetic analysis

## Abstract

Bovine babesiosis has substantial economic implications in the cattle industry, emphasizing the need for a thorough understanding of the genetic diversity of the causative apicomplexan pathogen. Although babesiosis has been extensively studied globally, the genetic diversity of *Babesia* species in Malaysian and Nigerian cattle remains unreported. This study aims to bridge this gap by detecting and characterizing *Babesia* species in selected cattle herds. Our investigation explores the genetic diversity of *Babesia* species in cattle from Selangor, Malaysia, and Ribah, Nigeria. Blood samples revealed a 32.9% infection rate via PCR analysis. Further genetic analysis detected variations in Malaysian *Babesia bigemina* isolates but genetic similarity among Nigerian isolates. Conversely, all *Babesia bovis* isolates displayed genetic homogeneity. In summary, this research identifies genetic diversity in *Babesia* species affecting Malaysian and Nigerian cattle, highlighting regional disparities.

## 1. Introduction

### Background 

*Babesia* infection seriously impacts the health and productivity of farm animals [1], often leading to profound loss of livelihood for many livestock owners [2].

*Babesia* species are prevalent in the tropics and subtropics [3], including Malaysia [4,5] and Nigeria [6,7]. The distribution of bovine babesiosis is related to the presence of the tick vector. In Asia, for example, where *Rhipicephalus microplus* originates, the tick vector has been distributed mainly with cattle to all continents [8] The tick is present in most of southern Asia and China and Central and South America, including Mexico, and it poses a major problem in Brazil [8]. *R. microplus*-infested cattle from Southern Asia introduced the tick to Madagascar and subsequently to other parts of Eastern and Southern Africa, affecting countries like South Africa, Zimbabwe, and Kenya and presently spreading at an alarming rate in West Africa, reaching part of central African countries [9].

Several outbreaks of bovine babesiosis, often with various fatality rates, have been reported in Malaysia since the year 1996 [4]; twenty-nine (29) outbreaks were recorded in 1996 [10], while fifteen (15) outbreaks with 17 cases and 17 deaths were recorded in 1997 [10]. In the year 1999, there were 15 outbreaks, with 11 cases and eleven deaths reported [10]. A spike of cases was observed in 2001, where 263 cases were recorded with eight (8) outbreaks [10]. In Nigeria, bovine babesiosis has been documented as far back as 1967 in the northwestern part of the country [11]. Kamani et al. [12] reported *Babesia* infection in cattle in some parts of northern Nigeria as the most common blood parasite observed. Lorusso et al., in 2016, also reported *Babesia* infection in cattle from north–central Nigeria. 

In nonimmune cattle, acute babesia infection causes massive intravascular haemolysis. It clinically manifests as fever, haemoglobinuria, anaemia, and jaundice [13]. When treatment is not instituted or delayed, the disease causes fatality in the affected herd [14]. These clinical manifestations, together with the evaluation of the haematological profiles of exposed/infected cattle, play a significant role in both diagnosis and treatment of the disease [15].

Nevertheless, many techniques have been used for the diagnosis of bovine babesiosis either severally or as a combination of two or more techniques. They begin with an earlier, less sensitive, and cumbersome microscopic examination of blood smears [16], then more sensitive serological methods that are also challenged by specificity issues [1]. Nowadays, numerous molecular techniques, including, e.g., polymerase chain reaction (PCR) [17] and restriction fragment length polymorphism (RFLP) [18] are used for the detection of *Babesia* infection in cattle. Genetic characterization of *Babesia* affecting cattle has also been made possible following sequencing analysis to explore their molecular diversity and evolutionary trends [19].

A few studies in Nigeria and Malaysia have explored the molecular epidemiology and diversities of cattle-infecting *Babesia* spp. Such information is critical to the diagnosis and or prevention of the disease. This study, for the first time, reports the genetic diversity, of cattle-infecting *Babesia* spp. in Selangor, Malaysia and Ribah, Kebbi State, Nigeria.

## 2. Methodology

### 2.1. Study Location 

Cattle used for this study were from selected herds located in Selangor, Malaysia (latitude 2°35′–3°60′ N and longitudes 100°45′–102°00′ E) [20] and Ribah, (latitude 11° N and longitude 5°and 6° E) Kebbi State, Nigeria [21]. Selangor is one of the states in Malaysia with the highest population of ruminant farmers. These farmers are engaged in both small ruminants as well as dairy and beef production using various management systems [22] while Ribah is known for its large livestock market in the state.

#### 2.1.1. Study Design

The study design was a cross-sectional study. A purposeful sampling method was adopted to target herds with a history of tick infestation in Selangor, Malaysia and Ribah, Kebbi State, Nigeria. Based on this criteria, selection of animals was performed.

#### 2.1.2. Selection of Animals

A total number of 85 (Malaysia N = 35, Nigeria N = 50) cattle were randomly selected for the study. Kedah-Kelantan x Brahman breed beef cattle of varying ages (1–11 years old) and sex (male and female) were selected from five different farms that had history of tick infestation in Selangor, Malaysia; each farm has an average of 50 cattle, with seven cattle selected from each of the farms.

Similarly, Sokoto Gudali breed beef cattle of varying ages (1–11 years old) and sex (male and female) were selected from five different farms with the same history of tick burden in Ribah, Kebbi State, Nigeria with ten cattle selected from each farm. The number of cattle per farm averaged 100. The animals were maintained in semi-intensive managements system in both locations.

The body condition score (BCS) of the animals was noted and grouped as A (BCS = 1–2) and B (BCS = 3–4) [23].

### 2.2. Sample Collection 

Ethical approvals (UPM/IACUC/AUP-R008/2020; UDUS/IACUC/2022/AU-R0-2) for the collection of blood samples from cattle at the respective locations was granted in both countries. The collection of samples was performed according to the approved guidelines contained therein. The cattle farms owners consented to this study with a written consent form.

Five mL of blood was collected aseptically from the jugular vein of each cattle using a blood vacutainer out of which three mL and two mL from each collected sample were placed into a different *Eethylenediaminetetraacetic acid* (*EDTA*) tube [24] to be used for microscopic detection of *Babesia* spp. and PCR detection of *Babesia* spp., respectively. Each sample was adequately labelled and repeatedly inverted a few times to mix and then placed into an icebox containing ice packs. The samples were then transported to the Haematology and Clinical Biochemistry Laboratory, Faculty of Veterinary, Medicine, University Putra Malaysia, and Clinical Pathology Laboratory Faculty of Veterinary Medicine and/or Centre for Advanced Medical Research and Training (CAMRET), Usmanu Danfodiyo University Sokoto, Nigeria, respectively, for further analysis.

### 2.3. Thin Blood Smear Examination 

A thin blood smear was performed to detect *Babesia* spp. microscopically, as described by [14].

### 2.4. PCR and Nested PCR Detection of Babesia spp.

#### 2.4.1. DNA Extraction 

Extraction of the genomic DNA from blood samples collected from the Malaysian Cattle was carried out at the Haematology and Clinical Biochemistry Laboratory Faculty of Veterinary Medicine University Putra Malaysia. The genomic DNA was extracted from blood samples from Nigerian cattle at the Centre for Advance Medical Research and Training, College of Health Sciences Usmanu Danfodiyo University Sokoto. The extraction was done using QIAGENE and DAAN Gene commercial extraction kits according to the manufacturer’s instructions for Malaysian and Nigerian cattle, respectively. The extracted DNA samples were stored at −20 °C until further analysis.

#### 2.4.2. Optimization of PCR Conditions

Polymerase chain reaction analysis for detection of *Babesia* spp. was performed using a commercially available optimized kit (AccuPower^®^ Babesia PCR kit by BIONEER, Bioneer Global Center, 71, Techno 2-ro, Yuseong-gu, Daejeon 34013, Republic of Korea) However, some optimization of the PCR conditions for specific detection of the 412 bp gene fragment of *B. bigemina* rap-1a and the 503 bp gene fragment of *B bovis* sbp-4 was carried out according to the steps recommended by [25] with some modifications.

#### 2.4.3. Detection of *Babesia* Species by PCR and Nested PCR

Commercial AccuPower^®^ *Babesia* PCR kit by BIONEER was used according to the manufacturer’s instruction to detect the presence of a 932 bp fragment of 18S rRNA gene of *Babesia* spp. from the DNA samples in both countries. Briefly, for each of the samples, 17 µL of DEPC (diethyl pyrocarbonate)-distilled water was added to the PCR premix tube followed by 3 µL of DNA template or 3 µL of the positive control, respectively. In contrast, 20 µL of DEPC-distilled water was added to one of the PCR premixes tubes as a negative control. All positive samples from this PCR reaction were then used for specific detection of *B. bigemina* and *B. bovis* using specific primers for their respective target genes (Table 1). The thermocyclic conditions for the PCR reaction are shown in Table 2.

From the 18S rRNA *Babesia* spp.-positive samples from the Malaysian cattle, initial PCR amplification was done using sets of specific primers (Table 1) targeting Rhoptry Associated Protein-1A (RAP-1A) of *B. bigemina* and Spherical Body Protein-4 (SBP-4) of *B. bovis*, respectively. Initial PCR amplification was performed using Bioline^®^ UK (by Biotechnology company, London, UK) PCR kit in a 25 µL volume reaction containing 12.5 µL of my taq red mix, 1 µL of each of the forward and reverse primer, 4 µL of DNA template, and 6.5 µL of distilled water for molecular biology. No DNA template was added to the negative control tube; instead, 10.5 µL of distilled water was added. A nested PCR was followed using a set of nested primers each (Table 1), while 4 µL of the amplicons generated from the first amplification was used as the template.

Specific PCR amplification was also performed on the 18S rRNA *B.* spp.-positive samples from Nigerian cattle using a commercial PCR kit (BIONEER) in a 20 µL volume reaction mixture according to the manufacturer’s instruction with slight modification. Briefly, initial amplification was done using 10 µL of master mix cocktail, 1 µL each of both forward and reverse primer, 4 µL DNA template and 4 µL of water for molecular biology except for the negative control, where 8 µL of water was added instead. A nested PCR reaction was carried out using 10 µL of the master mix cocktail, 1 µL each of forward and reverse primer, 2 µL of the amplicons from the initial amplification (as template), and 6 µL of water for molecular biology except for the negative control, where 8 µL of water was added. 

Amplicons generated from the PCR and nested PCR reaction were subjected to gel electrophoresis using a 2% agarose gel with 5 µL of redsafe nucleic acid staining solution at 70 volts for 75 minutes. A total of 5 µL of 100 bp DNA ladder was placed in one or two of the wells to enable detection of the size of the amplified DNA. Amplified target DNA was visualized under UV illumination using gel documentation [28].

#### 2.4.4. Sequencing and Bioinformatics Analysis 

Amplicons generated from three of the *B. bigemina* RAP-1A nested PCR-positive samples isolated from Nigerian cattle and nine from the Malaysian cattle, together with amplicons generated from all the four *B. bovis* SBP-4-positive samples from Malaysian cattle, were selected and sent for sequencing via the Sanger sequencing method using their respective specific nested PCR primers (Table 1). The obtained sequences were subjected to a BLAST search on the BLASTn tool of the NCBI website (https://blast.ncbi.nlm.nih.gov/Blast.cgi (accessed on 18 December 2021)). The correct species identity was inferred by comparing the query sequences with those found on the GenBank database, and a 100% identity match with the homologues on the GenBank confirms the species. The obtained sequences were eventually submitted to GenBank and are retrievable under the assigned accession numbers (Table 3). A phylogenetic tree was constructed using the Kimura two-parameter model [29] and neighbor-joining method option among the *B. bigemina* sequences generated from this study, this is because of its speed and scalability especially with the larger data set involved [30] and its robustness to data errors compared to other options e.g., maximum likelihood option which was used among the *B. bovis* sequences generated in this study. In both cases, selected reference sequences from GenBank were added. Tree construction was achieved using MEGA X version 11 software. The process involves a clustalW alignment of the detected respective gene sequences with some reference sequences on Genbank, which were stored in FASTA format following editing of the detected gene sequence using bioedit version 7.2 software. Further trimming of the sequences was then performed on MEGA X software from both the 3′ and 5′ ends in such a manner that ensures greater continuity of the aligned sequences on both ends. A final size of 291 bp RAP-1A gene fragment for *B. bigemina* and 364 bp SBP-4 gene fragment for *B. bovis* sequences was achieved and these were both used for the respective tree construction which follows a test for the best fit model carried out on MEGA X software. This was then followed by tree construction using the methods mentioned above.

### 2.5. Statistical Analysis

Data analysis was performed using SPSS version 25 software, and data are expressed as mean ± standard error. The association between BCS and the presence of *Babesia* spp. infection, was determined using chi-square [24,31], and statistical significance was inferred at *p* < 0.05.

## 3. Results

### 3.1. Microscopic Examination of Thin Blood Smear

All the samples evaluated by thin blood smear examination revealed negative for *Babesia* spp. in both selected herds.

### 3.2. Molecular (PCR and Nested PCR) Detection

This study revealed a total of twenty-eight (28) *Babesia* spp.-positive samples out of the 85 samples evaluated, representing a 32.9% infection rate.

#### 3.2.1. PCR Detection of *Babesia* Species 

Polymerase chain reaction analysis of 18S rRNA of *Babesia* spp. revealed an expected fragment size of 932bp in nine (9) out of a total of thirty-five (35) samples, representing an infection rate of 25.7% among the sampled Malaysian cattle. Similarly, nineteen (19) (38%) out of fifty (50) sampled Nigerian cattle were shown to be positive.

#### 3.2.2. Nested PCR Detection of *B. bigemina* and *B. bovis*

Specific PCR detection using primers (Table 1) targeting 879 bp gene fragment of *B. bigemina* Rhoptry Associated Protein 1-A (RAP-1A) and 907 bp gene fragment of *B*. *bovis* Spherical Body Protein-4 (SBP-4) following initial amplification of all the 18S rRNA *B.* spp.-positive samples in both Malaysia and Nigeria revealed no visible band on gel.

However, nested PCR using the specific nested primers targeting 412 bp gene fragment of RAP-1A and 503 bp gene fragment of SBP-4 respectively, reveal nine (9) *B. bigemina* positives out of nine (9) 18S rRNA *B.* spp. positives earlier detected from the Malaysian cattle, representing a 100% *B. bigemina* infection rate and four (4) *B. bovis* positives out of the nine (9) 18S rRNA, representing a 44.4 % *B. bovis* infection rate.

From the selected Nigerian cattle, a nested PCR targeting the 412 bp gene fragment of RAP-1A of *B. bigemina* reveal four (4) positives out of a total of nineteen (19) 18S rRNA *B.* spp. positives earlier detected. This represents a 21.1% *B. bigemina* infection rate, while no band was seen on gel following nested PCR targeting 503 bp gene fragment of SPB-4 of *B. bovis,* representing a 0% *B. bovis* infection rate.

#### 3.2.3. Phylogenetic Analysis

**Figure 1:**
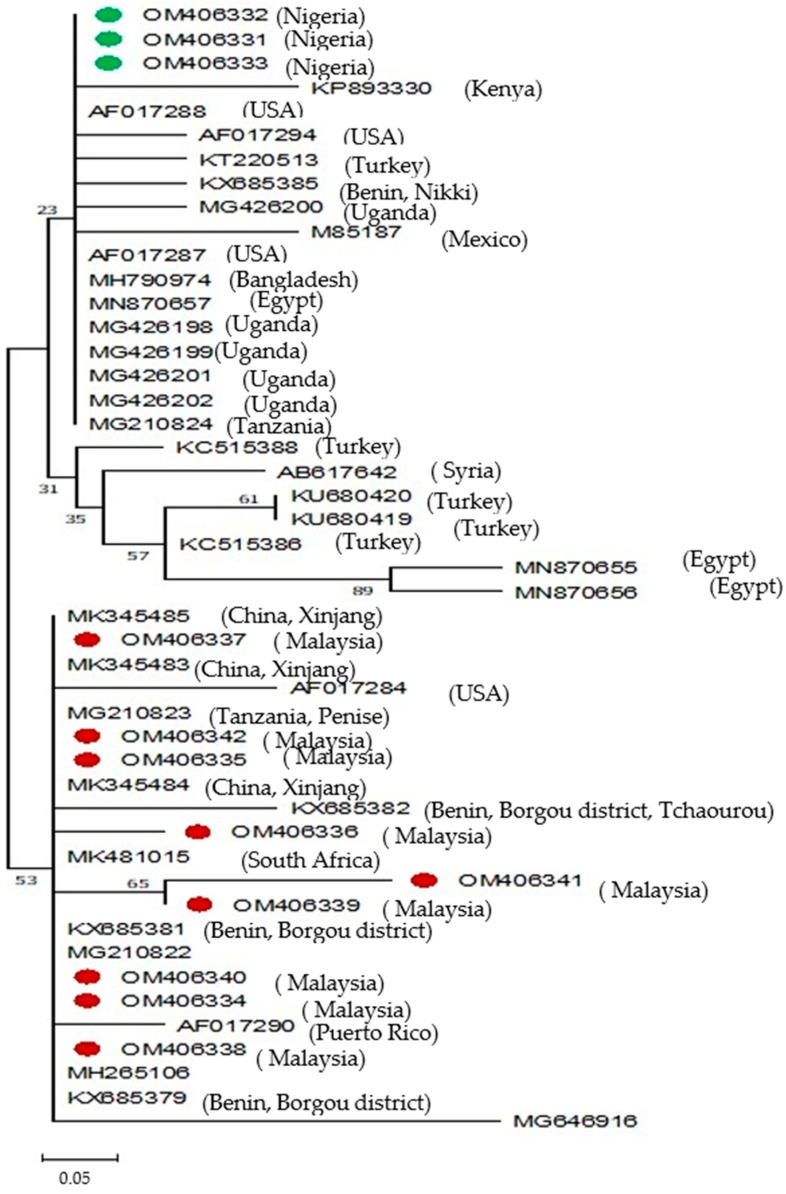
Phylogenetic tree of *B. bigemina* isolates obtained from Malaysian and Nigerian Cattle based on RAP-1A gene sequences. The evolutionary history was inferred using the neighbor-joining method [30]. The optimal tree is shown. The percentage of replicate trees in which the associated taxa clustered together in the bootstrap test (500 replicates) are shown next to the branches [32]. The tree is drawn to scale, with branch lengths in the same units as those of the evolutionary distances used to infer the phylogenetic tree. The evolutionary distances were computed using the Kimura two-parameter method [29] and are in the units of the number of base substitutions per site. This analysis involved 47 nucleotide sequences. Codon positions included were 1st, 2nd, 3rd, and noncoding. All ambiguous positions were removed for each sequence pair (pairwise deletion option). There were a total of 337 positions in the final dataset. Evolutionary analyses were conducted in MEGA11 [33]. Sequences determined in this study are indicated in green for Nigerian isolates and red for Malaysian isolates.

**Figure 2:**
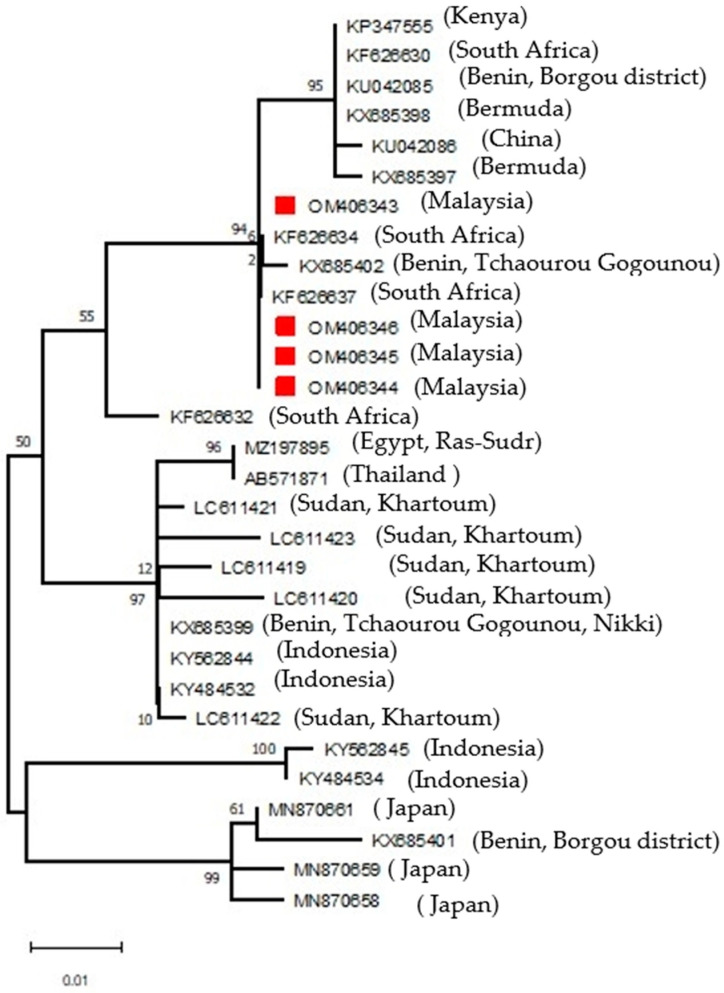
Phylogenetic tree of *B. bovis* isolated from Malaysian cattle based on the SBP-4 gene. The evolutionary history was inferred by using the maximum likelihood method and the Kimura two-parameter model [29]. The tree with the highest log likelihood (−939.85) is shown. The percentage of trees in which the associated taxa clustered together is shown next to the branches. Initial tree (s) for the heuristic search were obtained automatically by applying neighbor-join and BioNJ algorithms to a matrix of pairwise distances estimated using the maximum composite likelihood (MCL) approach and then selecting the topology with superior log likelihood value. A discrete gamma distribution was used to model evolutionary rate differences among sites (5 categories (+*G*, parameter = 0.2145)). The tree is drawn to scale, with branch lengths measured in the number of substitutions per site. This analysis involved 30 nucleotide sequences. Codon positions included were 1st, 2nd, 3rd, and noncoding. There was a total of 364 positions in the final dataset. Evolutionary analyses were conducted in MEGA11 [33]. Sequences determined in this study are indicated in red.

Phylogenetic trees of the sequenced isolates of *B. bigemina* RAP-1A gene and *B. bovis* SBP-4 gene are presented in Figure 1 and Figure 2, respectively.

#### 3.2.4. *B. bigemina* Phylogenetic Tree

The *B. bigemina* phylogenetic tree based on the RAP-1A gene is presented in Figure 1 below.

All the RAP-1A gene sequences from Nigerian isolates (OM406331, OM406332, OM406333) belong to the same clade and have an immediate common ancestor. They also share a common ancestor and clade together with isolates from Kenya (KP893330), Uganda (MG426200), Mexico (AF012788), and Bangladesh (MH790974). Seven (7) Malaysian isolates (OM406334, OM406335, OM406336, OM406337, OM406338, OM406340, and OM406342) clade together; they have an immediate common ancestor, which is ancestor to the other two (2) Malaysian isolates (OM406339 and OM406341) that also have an immediate common ancestor and belong to the same subclade. The seven isolates also share a common ancestor with isolates from Xinjian, China (MK355485), Pakistan (MG646916) Philippines (MH265106), and South Africa (MK481015).

#### 3.2.5. *B. bovis* Phylogenetic Tree

The *B. bovis* phylogenetic tree based on the SBP-4 gene sequences is presented in Figure 2 below.

The SBP-4 gene sequences from Malaysian isolates (OM406343, OM406344, OM406345, OM406346) (shown in red) belong to the same clade and share an immediate common ancestor. They also share a common ancestor with the common ancestor of the isolates from South Africa (KF626632) and Benin (KX685402), both of which have the same immediate common ancestor and belong to the same subclade.

#### 3.2.6. Estimates of Evolutionary Divergence between Sequences Based on the RAP-1A Gene of *B. bigemina* Isolates

A genetic distance of 0.0% was observed among the sequences of Nigerian *B. bigemina* isolates (OM406331, OM406332, OM406333) based on the RAP-1A gene sequences. Similarly, genetic distances of 0.0% were seen among six (OM406334, OM406335, OM406337, OM406338, OM406340, and OM406342) out of nine Malaysian isolates. However, a 0.3% distance was seen among two (OM406336 and OM406339) out of nine Malaysian isolates with the six isolates. These two isolates, however, have a 0.0% genetic distance between them. One Malaysian isolate (OM406341) showed a genetic distance of 0.1% with the six similar isolate and 1.2% with the two similar isolates. There were genetic distances of 0.2%, 0.4%, and 0.6% between sequences of the Nigerian isolates and those of six similar (OM406334, OM406335, OM406337, OM406338, OM406340, and OM406342), two similar (OM406336, OM406339), and one (OM406341) Malaysian isolate(s), respectively.

#### 3.2.7. Estimates of Evolutionary Divergence between Sequences Based on the SBP-4 Gene of *B. bovis* Isolates

There was no (0.0%) genetic distance observed among the Malaysian *B. bovis* sequences based on the SBP-4 gene. However, the Malaysian sequences showed some genetic distances of 0.8%, 3.2%, 4.1%, and 4.8% between them and the isolates from Kenya (KP347555), Benin (KX685399), Egypt (MZ197895), and Indonesia (KY562845), respectively.

### 3.3. Babesia Infection Status among the Selected Cattle and Body Condition Score

The association between the body condition score of the *Babesia.* spp. infected and non-infected among the Malaysian and Nigerian cattle was analyzed and presented in Table 4, bellow.

## 4. Discussion, Conclusions and Recommendations

### 4.1. Discussion

Previous studies, including a serological survey by [4] and a molecular survey [5], reported *Babesia* infections in cattle from Selangor, Malaysia, while *Babesia* infection in cattle from Ribah, Kebbi State, Nigeria has not been previously reported. Herein, we report the molecular detection of *B. *bigemina** and *B. bovis* from cattle in Selangor Malaysia and in Ribah, Kebbi State, Nigeria. We also, for the first time, explored the phylogeny of the detected *Babesia* isolates from both locations.

Microscopic examination of thin blood smears in both study locations was negative for the *Babesia* parasite. This was contrary to the findings of [34,35]. In the current study, the negative microscopy observed could be due to low parasitaemia and suggestive of carrier state infection [36,37]. Indeed, given the low sensitivity of conventional microscopic technique in the haemoprotozoan diagnosis, it is possible to obtain false positives, as reported by [38], who observed that cattle blood samples that tested negative for *B. bigemina* and *B. bovis* on microscopic examination of thin blood smears were actually positive when screened using nested PCR.

In this study, the total PCR detection rate of 25.7% infection in Malaysian cattle from Selangor was lower than the reported 30.5% reported by [39]. The higher rate observed by Ola-Fadunsin, however, can be attributed to a variation in sample size and study locations. Whereas the later was a prevalence study and covered the entire 11 states of peninsula Malaysia including Selangor, the current study (aimed at detecting *Babesia* sp. within the cattle population in Selangor for characterization of some targeted gene fragments) purposely sampled a few farms within Selangor only. This study, however, revealed a higher rate when compared to the serological survey by [4]. Similar findings were reported by [40], who reported a higher *B. bovis*-positive nested PCR detection rate than ELISA detection rate in cattle from Jombang, Lamongan, Bulukumba, and Lombok Timur, Indonesia.

Results obtained in this study also revealed a 38% detection rate for *B*. spp. in Ribah, Kebbi state, Nigeria. This is much higher than the results obtained in the neighbouring north-central region by [7]. Also, the higher rate of *B. bigemina* infections observed in the current study locations (Selangor and Ribah) is indicative of a higher tick infection rate and transmission rate for *B. bigemina* as opposed to *B. bovis*, consistent with the findings by [7,41,42]. Tick species competition limiting the distribution of *R. microplus*, especially in Africa [13] might also explain the higher *B. bigemina* infection rate as compared to *B. bovis* observed in Ribah. However, the serological survey by Rahman reported the same prevalence rate of *B. bigemina* and *B bovis* infection in cattle from Selangor, in contrast with the findings in this study. The discrepancy with the findings in the current study could therefore be due to the differences between the indirect fluorescence antibody technique (IFAT) and molecular detection methods, in which the former has been associated with cross-reactivity problems [37]. The appearance of the expected 412 bp RAP-1A gene fragment of *B. bigemina* and 503 bp SBP-4 gene fragment of *B. bovis*, on agarose gel following nested PCR analysis using amplicons generated from the initial PCR reaction, and the second pair of primers further confirms the higher detection sensitivity of nested PCR technique over the conventional PCR detection method. The former is handy in low parasitemias as in carrier state animals [17]. Similar findings were reported by [43] who reported a higher *B. bovis* detection sensitivity using nested PCR compared to the conventional PCR method.

In Ribah, *Babesia* spp. infection was detected in 19 (38%) out of 50 (100%) cattle tested. However, only 4 (21.1%) of these 19 were positive for the expected 412 bp RAP-1A fragment of *B. bigemina* and none (0%) to the expected 503 bp SBP-4 of *B. bovis.* This observation supports the possibility of endemic stability [44] for *B. bigemina* as against *B. bovis*, thereby leading to a higher transmission rate. Also, this finding supports a possible occurrence of other species of *Babesia* different from *B. bigemina* and *B. bovis* circulating among the cattle population in Ribah, which could be pathogenic [45], detected *B.* spp. Mymensingh in cattle from Sri Lanka when one of the cattle tested was positive for *Babesia* on a thin blood smear but appeared negative for *B. bovis*, *B. bigemina*, and *B. ovata* following nested PCR detection using respective specific primers. However, phylogenic analysis of the 18S rRNA and cytochrome oxidase subunit III gene sequences of the isolate revealed a positive result for *Babesia* spp. Mymensingh, which was not previously reported in Sri Lanka but was the sole isolate in a cow with clinical babesiosis.

Phylogenetic analysis of the *B. bigemina* isolates from Malaysian cattle based on the RAP-1 gene sequences revealed that six of the isolates are genetically conserved. Additionally, two isolates were found to be genetically conserved but 99.7% similar to the former. The remaining one isolate showed some polymorphism between the six similar isolates and two similar isolates, with 99% and 98% similarity, respectively. The three Nigerian isolates were found to be genetically conserved. They, however, showed some polymorphism with all the distinct Malaysian isolates.

The phylogenetic tree of the *B. bigemina* isolates in this study based on the RAP-1A gene sequences showed genetic diversity between the Malaysian isolates and the isolates from Nigeria. Thus, indicating the existence of genetic diversity among isolates from different geographical regions. *Babesia bigemina* isolates from Malaysia cluster differently from the Nigerian isolates. Interestingly, isolates from Malaysia were clustered together with other reference isolates from China (MK345484), the Philippines (MH265106), and Pakistan (MG646916), suggesting possible movement of trade animals with carrier infection and or infected *Boophillus* tick vector within the region [46]. However, an earlier detected *B. bigemina* isolate from South Africa (MK481015) cluster together with the Malaysian isolates and show a 100% similarity, suggesting a possible introduction to the naïve cattle in Malaysia from the infected South African carrier trade cattle.

The *B. bigemina* isolates from Nigeria cluster tightly together and clade together with isolates from Kenya (KP893330) [19] and Benin Republic (KX685385) [47]. A 99.4% similarity seen between the Nigerian and Kenyan isolates compared to the 99.7% similarity observed between Nigerian isolates and the isolate from Benin republic, indicating less genetic heterogeneity in isolates from Western Africa (Nigeria and Benin Republic) compared to isolates from Eastern Africa (Kenya). Isolates from Nigeria also clade together with an earlier detected isolate (AF017288) [48] from Mexico that is homologous (100% similarity) to the Nigerian isolates, thereby suggesting a possible introduction of *B. bigemina* infection to the naive Nigeria cattle from the infected cattle from Mexico. Indeed, there are records of the importation of trade animals, including cattle, into Nigeria from Mexico [49]; in the year 2018, for example, the Akwa Ibom State government was said to have imported cattle from Mexico [50].

The phylogenetic analysis of all the *B*. bovis isolates from Selangor Malaysia detected in this study, based on the SBP-4 gene sequences, showed that all the isolates clade closely together and are 100% similar. They also clade with an earlier detected reference isolate from South Africa (KF626634), which is a homologue, suggesting a possible introduction to the region and to the Malaysian herd from infected carrier cattle from South Africa via international trade in cattle [47]. In 2006, for example, the Malaysian government import quota liberalization policy aimed at enhancing self-sufficiency, especially for beef, and live animals (cattle and buffalo) were top on the list for agricultural products [51]. Other reference isolates cladding far away from the Malaysian isolates, for example, isolate (KX685401) from Benin republic, a west African country, exhibit some polymorphism, with 93.6% similarity to the Malaysian isolates, thus indicating genetic diversity from isolates from different geographical regions. Similar observations of the genetic diversity of *Babesia* spp. isolates as it relates to the geographical regions were reported by [19], who observed that *B. bigemina* isolates from Kenya were very closely related to the isolates from other African countries while *B. bovis* isolates differ significantly from the isolates from other regions.

Emaciation, anaemia, and hemoglobinuria are among the symptoms of bovine babesiosis [13]. Emaciated cattle have also been documented to have haemoglobinuria [52], which is a significant clinical finding in bovine babesiosis. In the current study, group A (BCS 1-2) cattle with poor body condition score from Nigeria show a significant association between body condition score and *Babesia* spp. infection. [31,52] also demonstrated that cachexation was linked to an increase in the loss of intact or lysed blood cells, which manifested as haemoglbinuria. These clinical manifestations support a possible occurrence of haemoprotozoan infection including babesiosis. Nevertheless, several other etiologies, e.g., malnutrition [53,54,55], neoplasia [56], and helminthiasis [57], have been associated with detrimental body conditions in many animals. Consistent with the findings in the current study as observed in Nigerian cattle, however, [55,56] reported haemoprotozoan infection in cattle with a low body condition score, while [58] demonstrated an association between occurrence of *Babesia bovis* in cattle with a poor body condition ratings.

### 4.2. Conclusions

The current study is likely the first report of the genetic characterization of the RAP-1A and SPB-4 genes of *Babesia* spp. in cattle from Malaysia and Ribah Nigeria. This study explored the phylogeny of detected *Babesia* spp. strains and revealed the existence of polymorphism among the *B. bigemina* strains detected from Selangor and homology among the *B. bigemina* strains detected from Ribah and *B. bovis* strains from Selangor. In the current study locations, different strains of *Babesia*. spp. were detected, and their phylogenies inferred based on the respectively targeted gene fragments. The current study therefore warrants the need for further investigation to understand how these disparities may impact disease prevalence, transmission dynamics, and possibly vaccine development in the respective regions.

### 4.3. Recommendations

Future epidemiological studies should be carried out to include a larger sample size and a wider study area to estimate the prevalence of *Babesia* infections not only in the selected states but the entire countries. The local transmission of *Babesia* spp., including the vector involved and the influence of seasonal variation and tick distribution on the occurrence of these pathogens, should also be investigated. It is also important that in Ribah, sequencing analysis of 18S rRNA *B. bigemina* positive samples should be considered in future studies. Similarly, in future molecular research, it is recommended to extend phylogenetic analysis to other genes with higher polymorphism such as the major surface antigen genes of *B. bovis*.

## Figures and Tables

**Table 1:** Sequences of primer sets used for detection of *Babesia* species.

Pathogen Target Gene	Assays	Primer Sequence (5′-3′)	Product Size (bp)	Reference
*Babesia* species 18S rRNA	PCR	*	932	[26]
*B. bigemina* RAP-1a	PCR	GAGTCTGCCAAATCCTTAC TCCTCTACAGCTGCTTCG	879	[27]
	Nested PCR	AGCTTGCTTTCACAACTCGCC TTGGTGCTTTGACCGACGACA	412	
*B. bovis* SBP-4	PCR	AGTTGTTGGAGGAGGCTAAT TCCTTCTCGGCGTCCTTTTC	907	[27]
	Nested PCR	GAAATCCCTGTTCCAGAG TCGTTGATAACACTGCAA	503	

* Primers incorporated in the premix tubes provided in the commercial kit, no sequence details provided.

**Table 2:** PCR Thermocyclic conditions.

Target Gene	Step	Temperature (°C)	Time	Cycles	Reference
*B. bigemina* 18S rRNA	Pre denaturation	95	5 min	40	[26]
	Denaturation	95	20 s		
	Annealing and extension	65	1 min		
	Final extention	72	5 min		
*B. bigemina* Rap-1a	Pre denaturation	95	5 min	40	[27]
	Denaturation	95	30 s		
	Annealing	58	1 min		
	Extension	72	30 s		
	Final extension	72	5 min		
*B. bovis* SPB-4	Pre denaturation	95	5 min	40	
	Denaturation	95	30 s		
	Annealing	58	1 min		
	Extension	72	30 s		
	Final extension	72	5 min		

Note: The same thermocyclic condition was used for nested PCR reaction for the same target gene.

**Table 3:** Accession numbers of DNA sequences deposited in GenBank for the *Babesia* species detected in this study.

Parasite Isolate	Target Gene	Accession Number	Sequence Length
*B. bigemina*	RAP-1A	OM406331	412
		OM406332	412
		OM406333	412
		OM406334	412
		OM406335	412
		OM406336	412
		OM406337	412
		OM406338	412
		OM406339	412
		OM406340	412
		OM406341	412
		OM406342	412
*B. bovis*	SPB-4	OM406343	503
		OM406344	503
		OM406345	503
		OM406346	503

**Table 4:** Showing association between *Babesia* infection status of cattle.

*Babesia* Infection Status Selangor	Body Condition Score (BCS) (1–2) (3–4)		Total
Infected	3 (8.5) ^a^	6 (17.6) ^a^	9 (25.7)
Not infected	15 (42.8) ^a^	11 (31.4) ^a^	26 (74.2)
Total	18 (51.4)	17 (48.5)	35 (100)
Ribah Infected	12 (24.0) ^a^	4 (8.0) ^a^	16 (32)
Not infected	13 (26.0) ^a^	21 (42.0) ^b^	34 (68)
Total	25 (50)	25 (50)	50 (100)

Note: Values in brackets are percentages, X2 = 2.125, df = 1; *p* > 0.05; values in columns with the same superscript did not differ significantly.

## Data Availability

DNA sequences obtained in this study have been submitted to GenBank database (accession number: OM406331, OM406332, OM406333, OM406334, OM406335, OM406336, OM406337, OM406338, OM406339, OM406340, OM406341, OM406342, OM406343, OM406344, OM406345 and OM406346).
